# Exploring the efficacy of various CNN architectures in diagnosing oral cancer from squamous cell carcinoma

**DOI:** 10.1016/j.mex.2024.103034

**Published:** 2024-11-05

**Authors:** Prerna Kulkarni, Nidhi Sarwe, Abhishek Pingale, Yash Sarolkar, Rutuja Rajendra Patil, Gitanjali Shinde, Gagandeep Kaur

**Affiliations:** a Department of CSE (AIML), Vishwakarma Institute of Information Technology, Kondhwa (Budruk) Pune, Maharashtra 411048, India; b CSE Department, Symbiosis Institute of Technology, Nagpur Campus, Symbiosis International (Deemed University), Pune, India

**Keywords:** Oral cancer histopathologic OSCC, Squamous cell carcinoma, Convolutional neural networks, Deep learning, Histopathological images, Convolutional Neural Networks

## Abstract

Oral cancer can result from mutations in cells located in the lips or mouth. Diagnosing oral cavity squamous cell carcinoma (OCSCC) is particularly challenging, often occurring at advanced stages. To address this, computer-aided diagnosis methods are increasingly being used. In this work, a deep learning-based approach utilizing models such as VGG16, ResNet50, LeNet-5, MobileNetV2, and Inception V3 is presented. NEOR and OCSCC datasets were used for feature extraction, with virtual slide images divided into tiles and classified as normal or squamous cell cancer. Performance metrics like accuracy, F1-score, AUC, precision, and recall were analyzed to determine the prerequisites for optimal CNN performance. The proposed CNN approaches were effective for classifying OCSCC and oral dysplasia, with the highest accuracy of 95.41 % achieved using MobileNetV2.

**Key findings:**

Deep learning models, particularly MobileNetV2, achieved high classification accuracy (95.41 %) for OCSCC.

CNN-based methods show promise for early-stage OCSCC and oral dysplasia diagnosis. Performance parameters like precision, recall, and F1-score help optimize CNN model selection for this task.

## Graphical abstract

**Figure:**
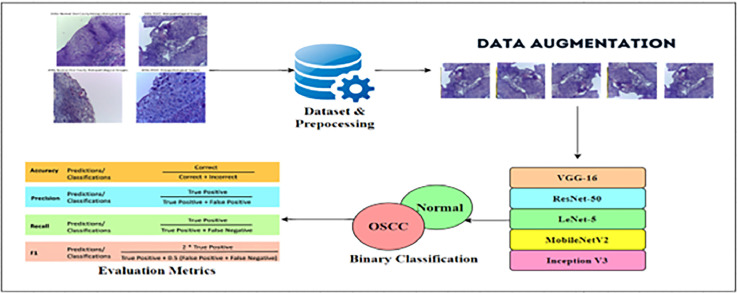

Specifications Table

**Table:** 

Subject area:	Medicine and Dentistry
More specific subject area:	Oral Squamous Cell Carcinoma
Name of your method:	Convolutional Neural Networks
Name and reference of original method:	• VGG16: [2] • ResNet50: [3] • LeNet5: [4] • InceptionV3: [5] • MobileNetV2: [6]
Resource availability:	Code (Github Link), Dataset

## Background

Oral cancer is a prevalent illness, frequently identified only in its advanced stages, which poses a significant challenge to effective treatment. >90 % of instances of oral cancer are of the most prevalent kind, oral squamous cell carcinoma (OSCC), which arises from the mucosal lining of the mouth [1]. Consequently, OSCC has been identified as a subtype of head and neck squamous cell carcinoma, the seventh most common cancer worldwide. It appears that head and neck cancer is a more complicated illness that needs highly specialized advice from oral and medical oncologists, radiologists, pathologists, and surgeons [7].

The development of oral cancer frequently results from exposure to carcinogens, mostly alcohol and tobacco, on the upper aerodigestive tract mucosal coverings. A sequence of events within the mucosa may result in premalignant and malignant lesions as a result of this exposure. It is interesting, nonetheless, that some people with oral cancer are not known to have used tobacco products or alcohol, nor do they display any other known risk factors.

The oral areas are home to neoplasms with a variety of cellular origins, such as salivary gland tumors, nasopharyngeal carcinoma, lymphomas, mucosal melanoma, and sarcomas. There are a number of rare histological variations of squamous cell carcinoma that affect prognosis and treatment options. Spindle cell or sarcomatoid squamous malignancies are less common in the oral cavity but more frequently seen on the lip and larynx.

Indeed, early detection is intrinsic to successful treatment and improvement in the outcomes of OSCC patients. Many forms of cancer are treatable or curable if treated early, and so many clinical protocols have been developed for the detection and staging of cancers [8].

So far, one considers a biopsy as the gold standard test to confirm the diagnosis of cancer[9]. Over the years, various research works have concentrated on combining AI with the improvement of medical diagnostics [10]. With the high consumption rate in diagnostic imaging guiding them, the research community has been well-positioned to investigate multiple applications in medical image analysis with substantive building blocks for further improvements in diagnostic accuracy and efficiency. Deep learning techniques, especially CNNs, have become very effective and powerful tools in analyzing medical images that diagnose oral cancer in squamous cells as compared to Machine Learning Algorithms [[11], [12], [13]]. Based on the large dataset for histopathological images, CNNs can learn complicated patterns and characteristics connected with cancerous cells with high accuracy and efficiency in identifying malignancies present in oral tissue samples [14]. This study focuses on the development and evaluation of algorithms for squamous cell images using CNNs with the aim to achieve much more accurate and improved results. The present study intends to propose a hybrid deep learning approach based on the power of CNNs for feature extraction so that the quality of oral cancer detection becomes more accurate and consistent.

## Method details

Deep learning is a subset of machine learning that normally involves learning depictions at distinct levels of hierarchy to allow construction of complex concepts [15]. When referring to techniques that enable computers to act like people, the term "artificial intelligence" is used generally. Regular pattern recognition involves identifying salient characteristics by careful inspection, which is followed by feeding the features into a simple neural network to perform sorting. Deep learning, on the other hand, exploits the system's ability to recognize significant features on its own to solve problems. Similar to a network of neurons, deep learning gathers input from the user and processes it via multiple layers to provide a response. One such deep learning algorithm is Convolutional Neural Networks. DCNNs are useful for learning the patterns from the images and are able to classify using these patterns, thereby eliminating the need for manual feature extraction[[16], [17], [18], [19]]. The present research is a comparative study of 5 Convolutional Neural Networks namely VGG-16, ResNet-50, LeNet-5, MobileNetV2 and Inception V3 [16].

**Fig. 1:**
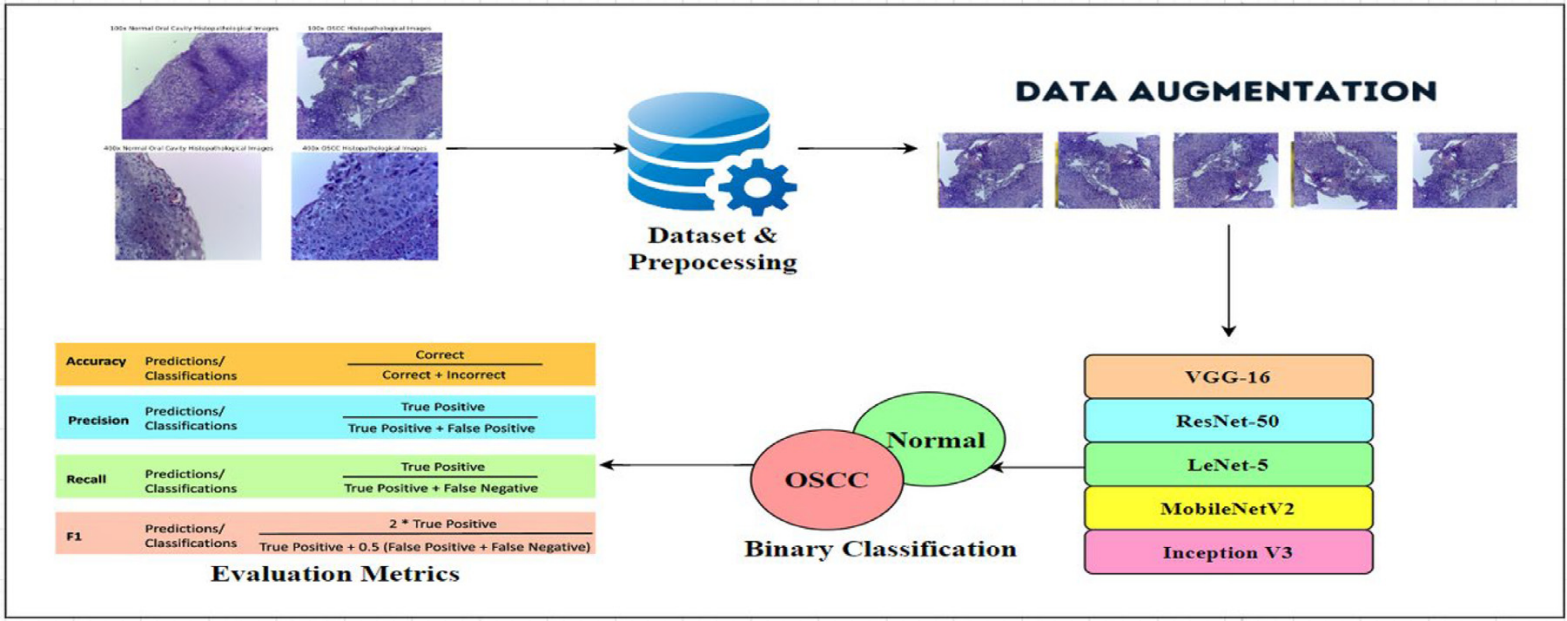
Structured workflow for binary classification of normal and OSCC images.

As shown in Fig. 1, the classification model predicts oral cancer in squamous cells using deep learning techniques such as VGG-16, ResNet-50, LeNet-5, MobileNetV2and Inception V3. The model is trained and tested on a labelled dataset that classifies the images into:

1. OSCC Histopathologic Image
2. Normal Cavity Histopathologic Image

**Fig. 2:**
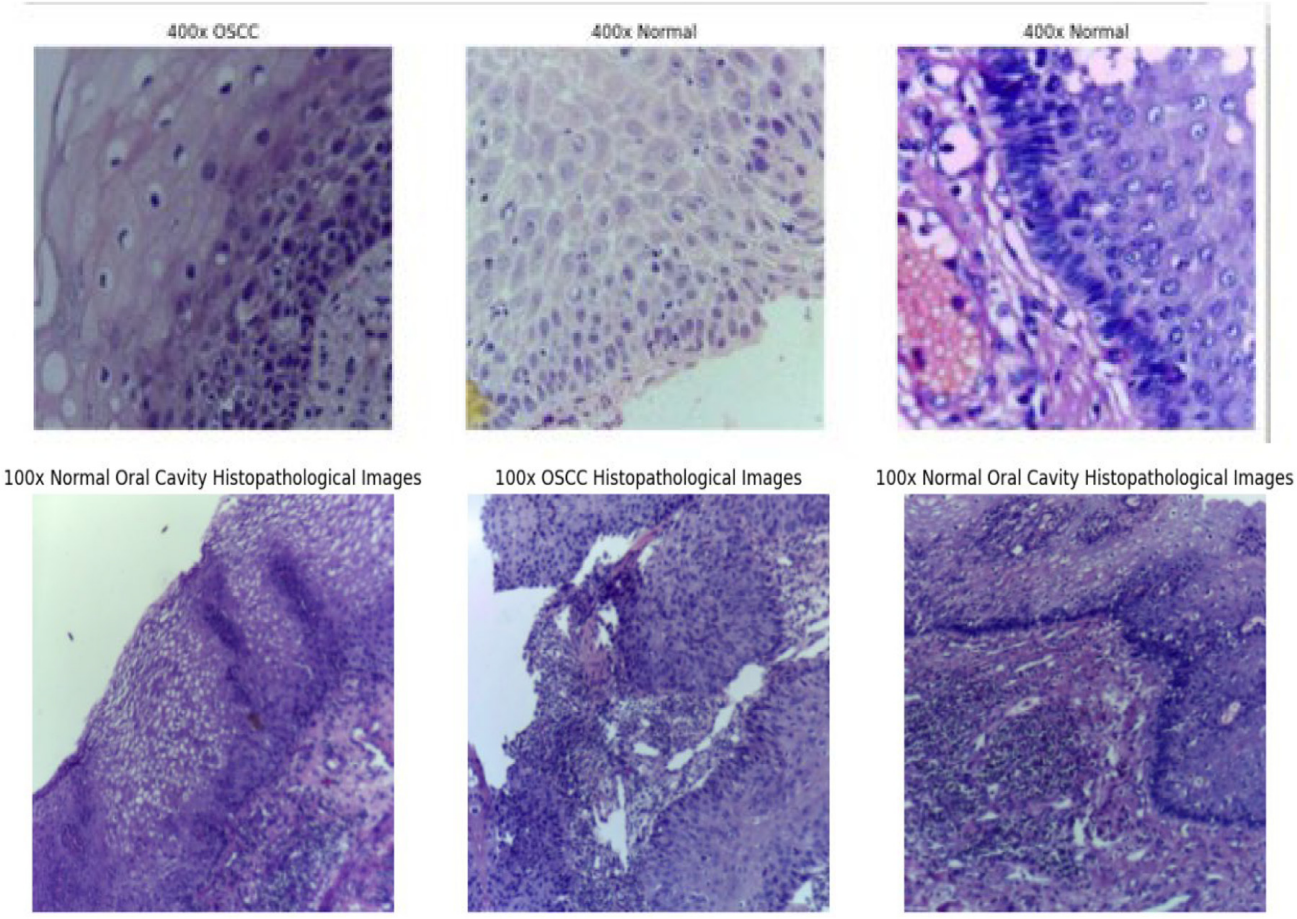
Different classes of histopathological images from the original dataset.

After classifying the images into OSCC Histopathologic Images and normal Cavity Histopathologic Images, Image processing is done to extract relevant information from this visual data. Fig. 2 shows different classes of histopathological images from the original dataset, highlighting the variations between OSCC and normal cavity images. The Fastai library based on PyTorch would provide a really great set of tools for creating and implementing fine-tuned image processing pipelines. These might include a number of processes that could involve augmentation, normalization, scaling, and modification. Image augmentation helps avoid overfitting by integrating techniques such as rotation, flipping, scaling, and color changes in the training dataset [20].

**Fig. 3:**
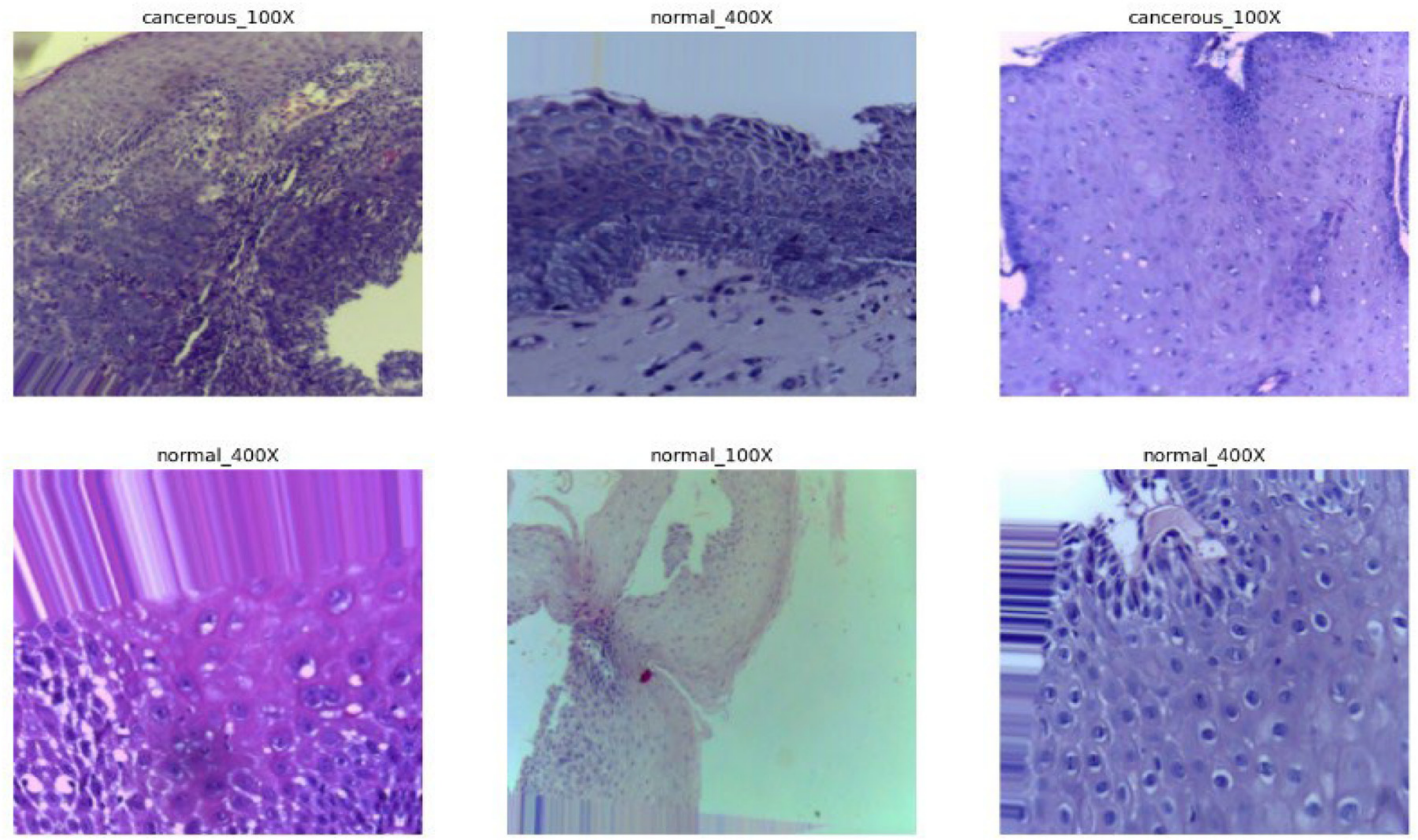
Images generated after augmentation of original dataset.

Fig. 3 shows images generated after augmenting the original dataset, demonstrating the variety introduced through augmentation techniques. A total of 6931 images were generated by augmenting the original dataset of 1224 images which was sourced from [21] with the help of the ImageDataGenerator class of keras. The images were rescaled by about 1/255, rotated randomly by about 0–40°, translated randomly by 20 % of height or width in either direction, randomly zoomed by 20 %, randomly flipped, and the newly created pixels were filled by the nearest pixel value.

Expanding the dataset helps deep learning models perform better and prevent overfitting by ensuring good model generalization [22]. Augmenting the dataset helps to expand size of the dataset without the necessity of gathering fresh data, as deep learning necessitates a significant amount of data. Table 1 shows the image details from the dataset in terms of type and quantity.

**Table 1:** Image details from dataset in terms of type and quantity.

Resolution	Class	Original number of Images	Number of Images after Augmentation
100X Magnification	Normal	89	1780
	OSCC	439	1615
400X Magnification	Normal	201	1815
	OSCC	495	1721

Consequently, this augmentation resulted in the division of the dataset into 80 % for training and 20 % for testing. In this work, four CNN architectures—VGG-16, ResNet-50, LeNet-5, MobileNet V2, and Inception V3—for the diagnosis of oral cancer will be implemented and evaluated using the Keras framework. The top layers of pre-trained models for VGG-16, ResNet-50, and Inception V3 were improved by using custom-made fully connected layers designed for the specific classification purpose. These models were further refined after initial training by freezing the base layers.

### VGG16 deep learning algorithm

**Fig. 4:**
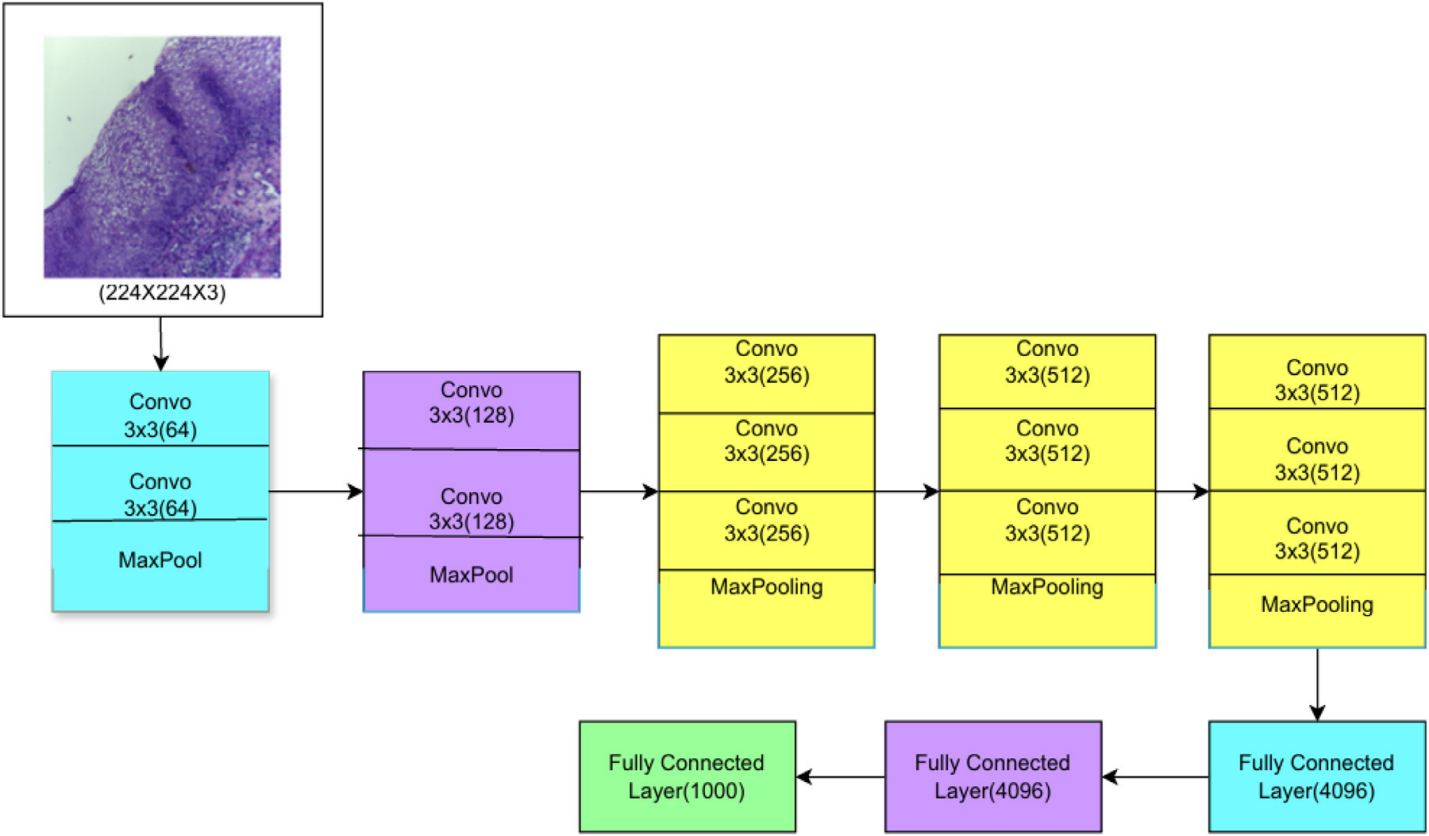
Architecture of VGG-16.

'VGG' refers for the University of Oxford's Visual Geometry Group, and the number '16′ in VGG16 denotes the network's 16 weighted layers. The deep convolutional neural network called VGG16 is employed in image classification. Fig. 4 shows the architecture of VGG-16, where the 16 layers of artificial neurons in the network analyze each image independently to improve prediction accuracy.. VGG16 incorporates maxpool layers with a 2×2 filter and a stride of 2, as well as convolution layers with a 3×3 filter and a stride of 1, that are in place of a large number of hyperparameters. In the subsequent stage, pre-trained weights are inserted into the VGG16 model to fine-tune it for data training.

The model's performance is assessed for each class, and errors are identified using performance metrics like as accuracy, recall, AUC, f1-score, and confusion matrix [23].

### ResNet-50 deep learning algorithm

**Fig. 5:**
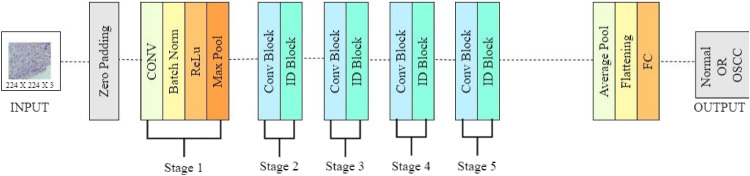
Architecture of ResNet-50.

ResNet-50, or Residual Networks, is a deep neural network used for many computer vision applications For eg; object detection and image segmentation. The topology, with residual connections, is very deep. This provides considerable mastering and subsequently turns the network into a very strong tool for classes of image classification tasks in medical imaging. Fig. 5 shows the architecture of ResNet-50, and the findings derived from the present study have emphasized the capacity of the model to support medical diagnosis and research, as pointed out herein below by its capability to distinguish between images with and without cancerous lesions [22].

The ResNet-50 model was imported with pre-training on weights on ImageNet and adapted for a binary classification assignment. Only the top layers were removed so that the network behaves like a feature extractor and allows only the last few layers to be trainable. Further, on top of the base model, a GlobalAveragePooling2D layer was added, followed by Dense layer with sigmoid activation for binary classification. The network was trained using the Adam optimizer, using a learning rate of 0.01 and a loss function of binary cross-entropy. The training lasted ten epochs, and the picture data creators provided both the training and validation datasets. Callbacks such as ReduceLROnPlateau and EarlyStopping were used to avoid overfitting and to dynamically adjust the learning rate in a more efficient way during training.

### LeNet-5 deep learning algorithm

**Fig. 6:**
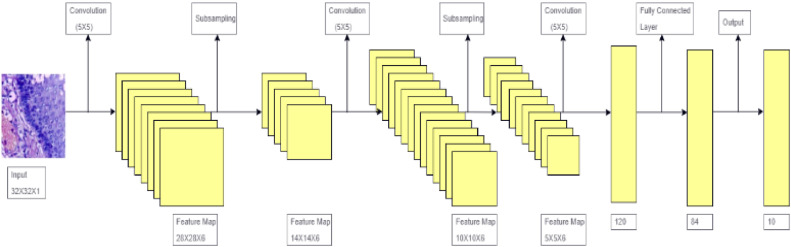
Architecture of LeNet-5.

LeNet-5, developed in 1998 by Yann LeCun and colleagues, was one of the earliest convolutional neural network (CNN) architectures. Fig. 6 shows the architecture of LeNet-5. Layers one to three are fully connected layers at the end, followed by two convolutional layers and subsampling (pooling) layers. The first layers in the architecture to apply various filters to extract feature maps are the convolutional layers. After that, these maps are down-sampled by average pooling to reduce dimensionality while keeping important details. The final fully linked layers divide the features into various groupings.

The MNIST dataset is a popular benchmark in the fields of computer vision and machine learning. It comprises of a vast collection of handwritten digit images that are often used to train and test convolutional neural networks (CNNs).

### MobileNetV2 deep learning algorithm

**Fig. 7:**
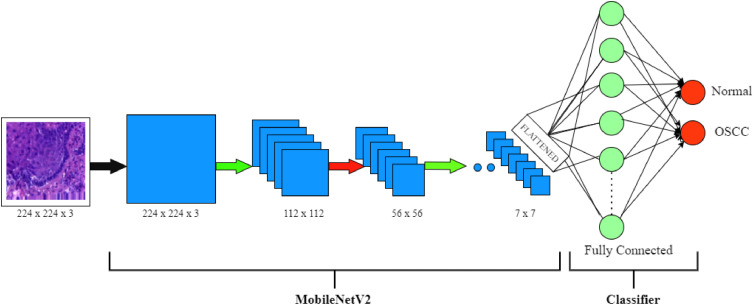
Architecture of MobileNetV2.

MobileNetV2 is an efficient, light-weight, yet powerful convolutional neural network model that has been designed to be deployed for mobile and embedded vision applications. Fig. 7 shows the architecture of MobileNetV2. Google's MobileNetV2 extends the success of its predecessor, MobileNetV1, by incorporating several state-of-the-art improvements contributing to its better performance and effectiveness.

Optimal performance in embedded and mobile applications is achieved with the MobileNetV2 architecture because of a well-chosen balance between efficiency and accuracy. First of all, the first convolutional layer down-samples an RGB image of the fixed size of 224×224 pixels and adds 32 channels after processing by the input layer. The basic building block of this system's architecture is the Inverted Residual Block. It includes depthwise convolution, projection layer-1×1 linear convolution, and expansion layer-1×1 convolution with ReLU6. These blocks are further facilitated by the shortcut connections that enhance the flow of a gradient especially when the dimensions of input and output are same. After these blocks, a 1×1 Conv2D layer extends channels to 1280, followed by a completely linked layer for classification and global average pooling.

### Inception V3 deep learning algorithm

**Fig. 8:**
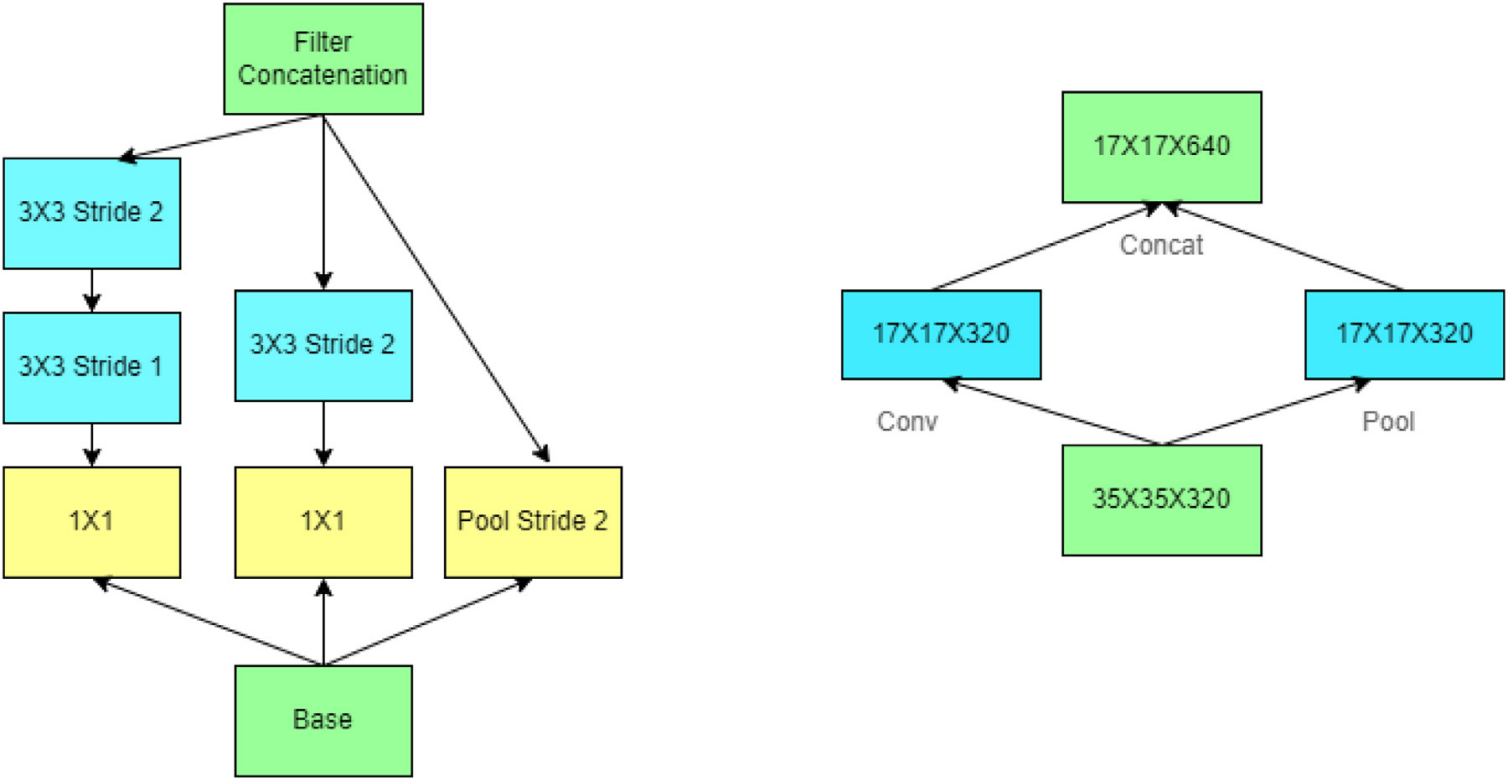
Architecture of inception V3.

To extract features from images, the InceptionV3 architecture employs a number of convolutional, pooling, and inception modules. Fig. 8 shows the architecture of InceptionV3. By employing filters of various sizes, inception modules—blocks of layers—enable the network to learn a range of features at various scales and resolutions.

Necessary libraries are imported for image processing (PIL) and for defining data configuration and transformations. Data configuration for the model is resolved and creates transformation functions based on the configuration. File path for an image is defined and loads the image using PIL, converting it to RGB format. Transformations to the image are applied to prepare it for input into the model.

For each image, it assumes the variable probabilities holds the probabilities for each image. It then prints the top 5 predicted categories along with their probabilities.

### Method validation

The pre-trained models VGG16 and ResNet50 beat LeNet5 and InceptionV3, suggesting that transfer learning is effective for OSCC image categorization. VGG16 and ResNet50 demonstrated superior precision, AUC, recall, and F1-scores, indicating their suitability for practical applications in medical image processing.

**Performance of VGG-16:**

**Fig. 9:**
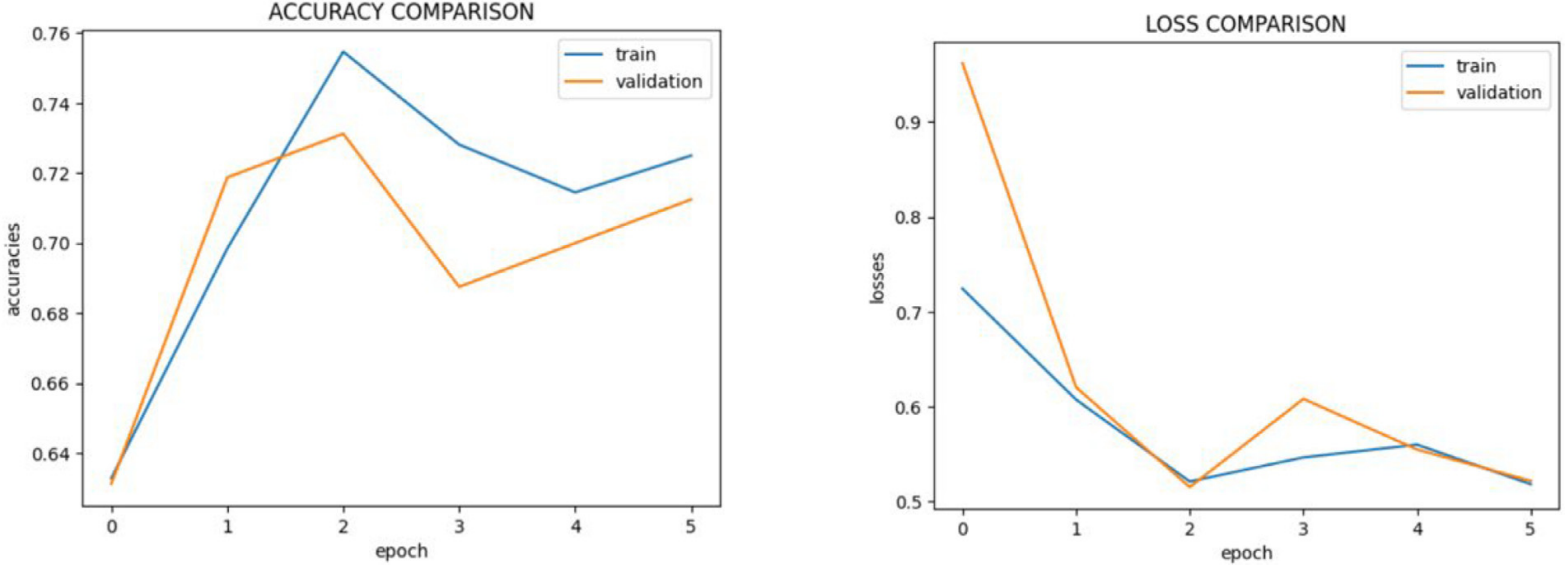
VGG-16 Model performance metrics.

A well-known convolutional neural network architecture, the VGG16 model, classified the dataset with an accuracy of 76.74 %. Fig. 9 shows the VGG-16 model performance metrics. This excellent performance shows that, in spite of any potential complications in the dataset, the model can learn and generalize features from the photos. The dense layers and narrow receptive fields of the model's design provide accurate feature extraction, which enhances its robustness.

**Confusion Matrix:**

**Fig. 10:**
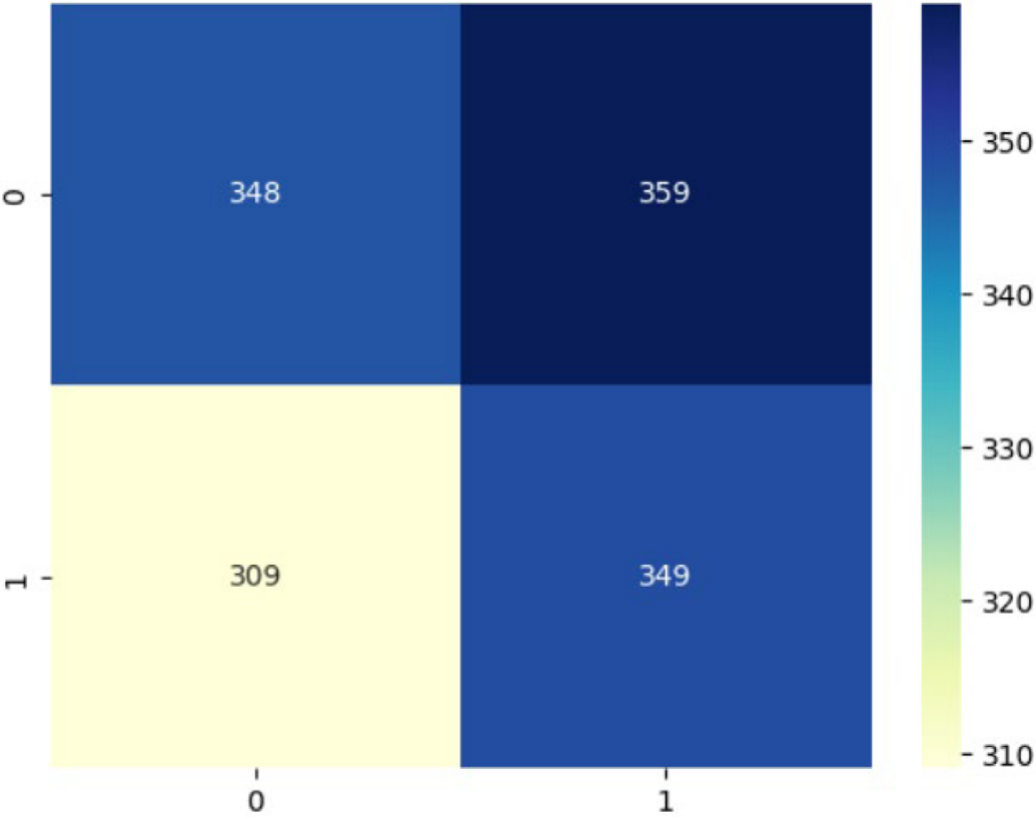
VGG16 model confusion matrix.

The generated confusion matrix indicates normal cells as 0 and Oral Squamous Carcinoma Cells as 1, thereby doing binary classification and generating a 2×2 matrix for VGG16 model. Fig. 10 shows the VGG16 model confusion matrix.

In the classification task, 348 normal cells were correctly classified as normal; there were 349 cancerous cells which were also correctly classified as cancerous. Other than this, there was some amount of misclassifications; 359 normal cells which were misclassified as cancerous, and 309 cancerous ones misclassified as normal.

By implementing some of the techniques like — balancing the dataset, adding more custom layers to the VGG16 model, modifying the loss function — the classification performance can be improved of the VGG16 model, thereby reducing misclassifications, particularly for cancerous cells.

**Performance of ResNet-50:**

**Fig. 11:**
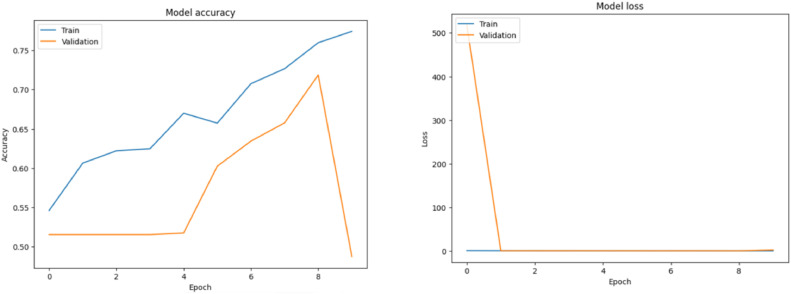
ResNet-50 model performance metrics.

Classifying photos into normal and malignant categories was accomplished with an accuracy of 71 % by the ResNet-50 model, which is renowned for its deep residual learning architecture. Fig. 11 shows the ResNet-50 model performance metrics. This outcome demonstrates the model's reliable operation and potent feature extraction skills. ResNet-50′s capacity to differentiate between complicated image classes in the dataset is demonstrated by its success.

**Confusion Matrix:**

**Fig. 12:**
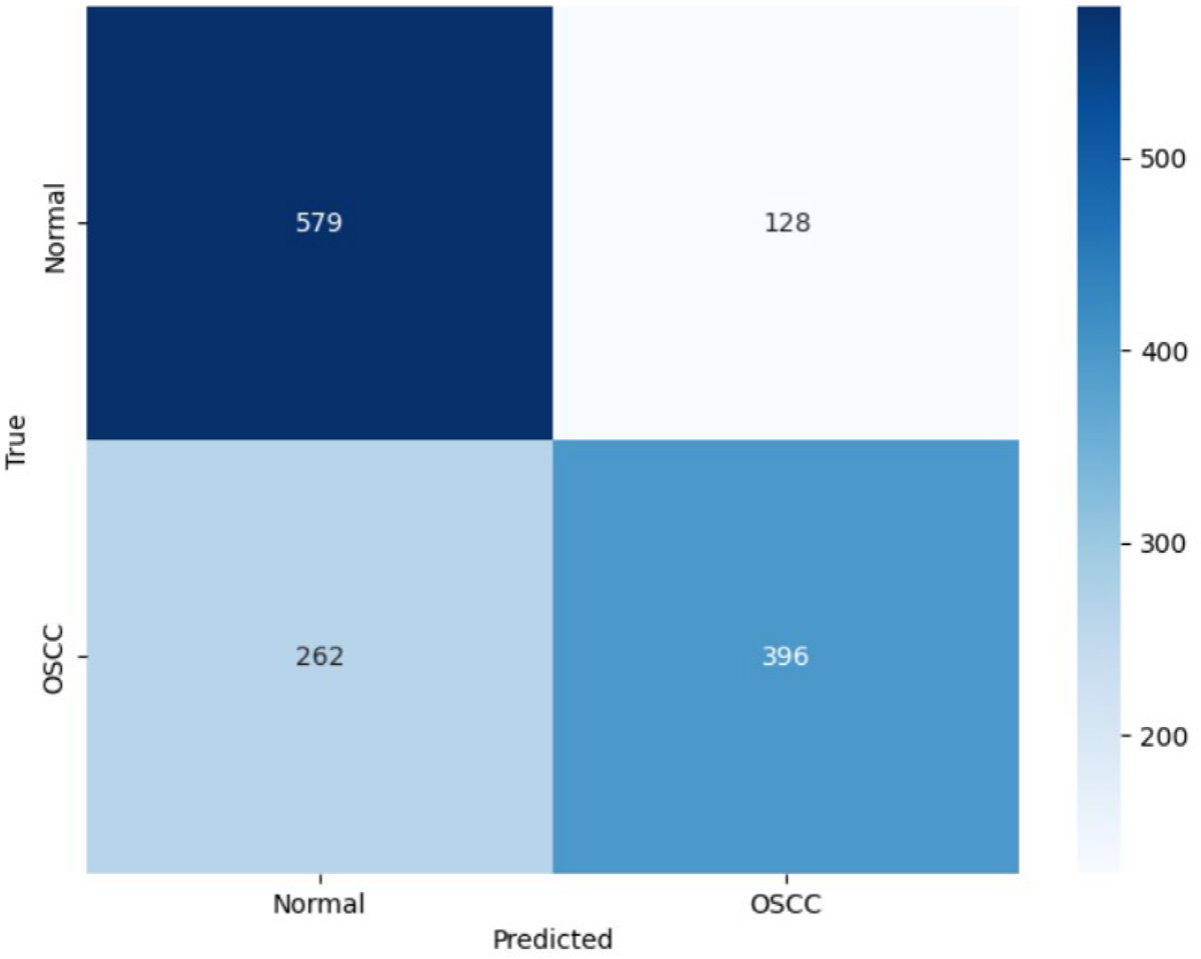
ResNet-50 model confusion matrix.

In that classification 579 normal cells and 396 cancerous cells are identified correctly. On the other hand, 128 normal cells are misclassified as well and reported as cancerous cells and vice versa, 262 cancerous cells are misclassified to be normal cells. Fig. 12 shows the ResNet-50 model confusion matrix.

True Negatives are also very high at 579, which means that the model is quite great at finding normally labelled cells. On the other hand, False Positives stand at 128, which are much lower compared to False Negatives 262; this means the model is more prone to missing a cancerous cell than wrongly labelling a normal cell as cancerous. A great solution to this can be implementing focal loss, which can help address the class imbalance by focusing more on hard-to-classify samples.

**Performance of LeNet-5:**

**Fig. 13:**
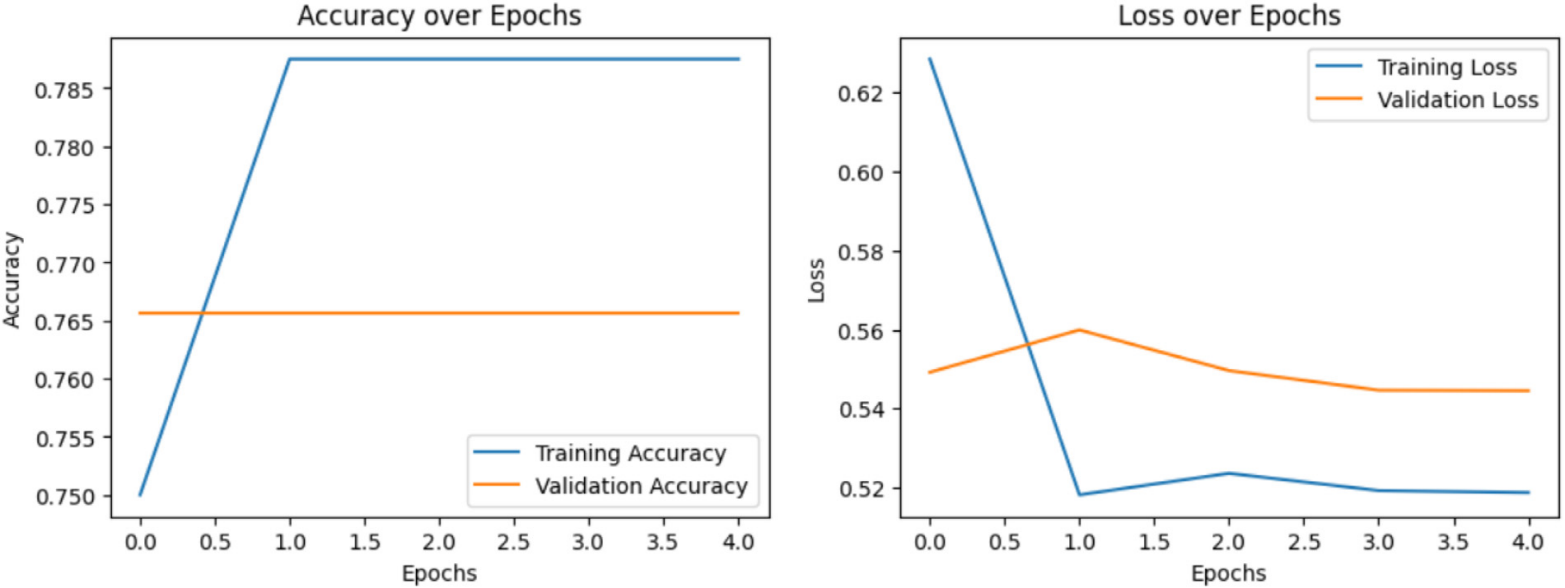
LeNet-5 model performance metrics.

The comparison investigation revealed that the LeNet-5 model is capable of achieving 76.54 % accuracy in picture classification tasks. Fig. 13 shows the LeNet-5 model performance metrics. However, when compared to other models such as VGG16 and ResNet-50, LeNet-5 demonstrated lower accuracy and feature extraction efficiency.

**Performance of MobileNetV2:**

**Fig. 14:**
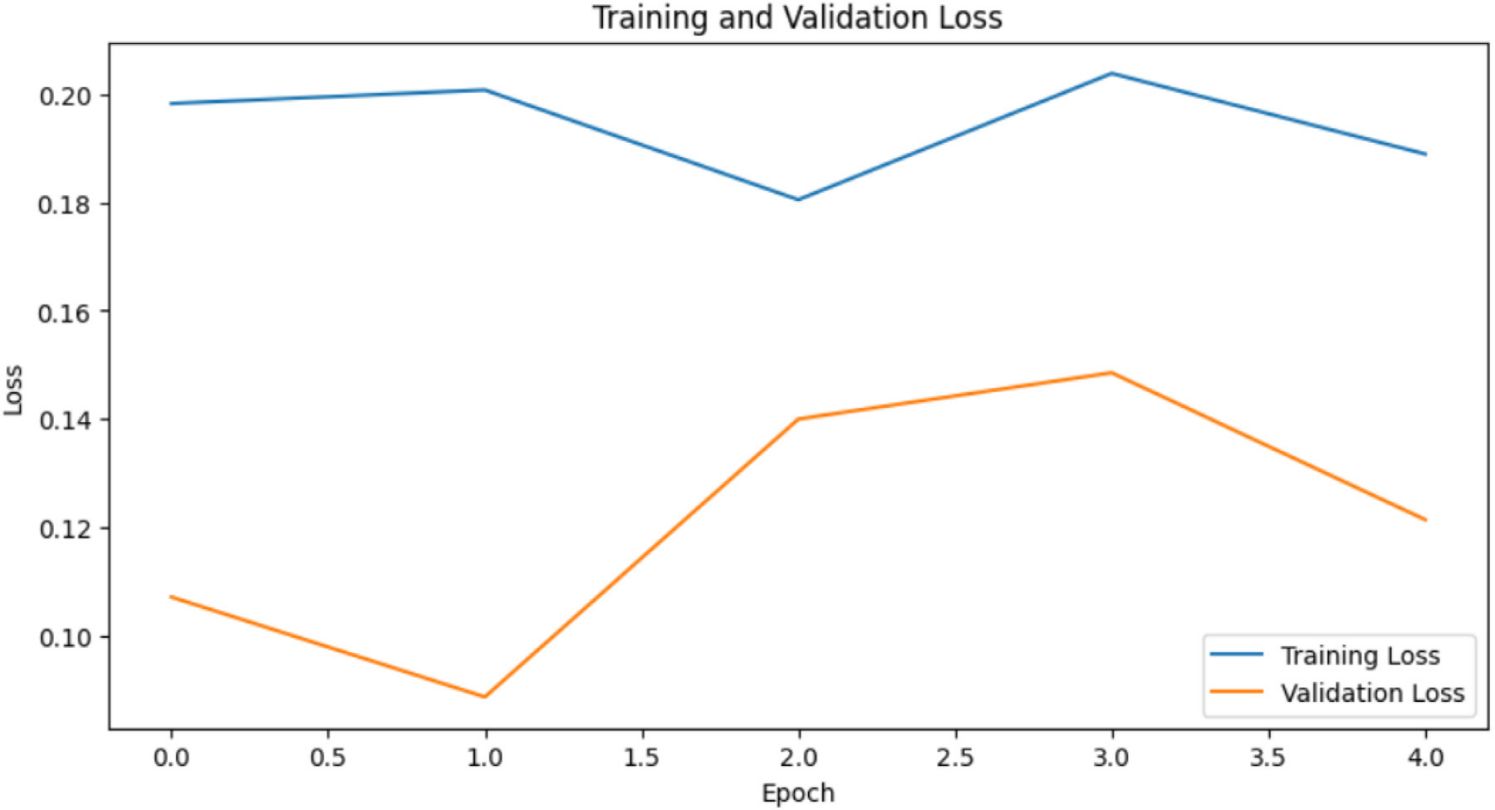
MobileNetV2 model performance metrics.

MobileNetV2 achieves an accuracy of 95.41 %, exhibiting robust performance metrics. Fig. 14 shows the MobileNetV2 model performance metrics. In order to acknowledge the variations in both training and validation loss over the epochs, emphasize that the model has shown good generalization capabilities, as revealed by the small difference between training accuracy and validation accuracy. The alignment of training accuracy and validation accuracy indicates that the model is not overfitting, despite these occasional oscillations. This great degree of accuracy is a result of the architecture's skill at striking a balance between computing efficiency and model complexity. This performance is enhanced by the inclusion of inverted residual blocks, which minimize the number of parameters while optimizing both depth wise separable convolutions and linear bottlenecks.

**Confusion Matrix:**

**Fig. 15:**
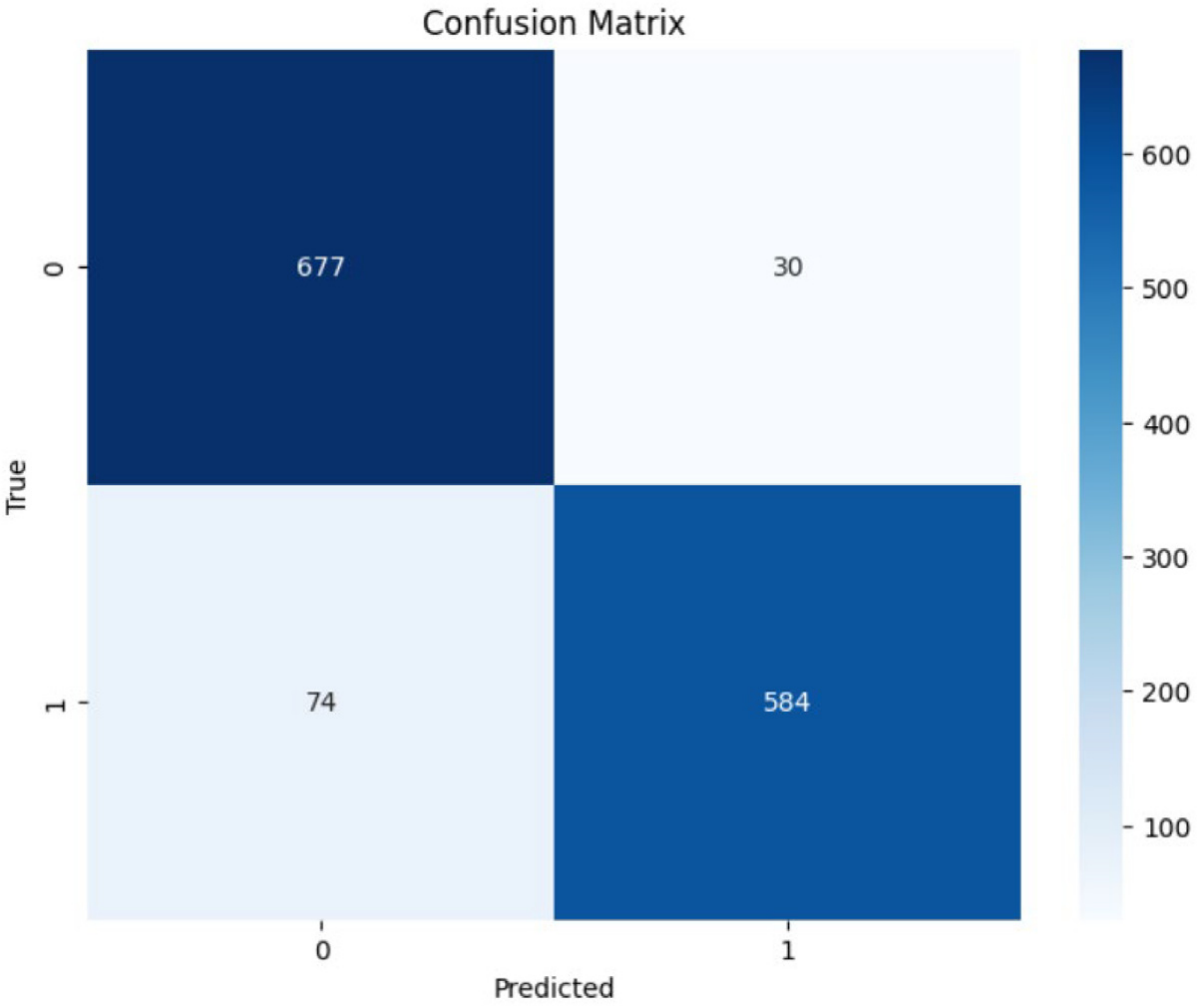
MobileNetV2 model confusion matrix.

The generated confusion matrix indicates normal cells as 0 and Oral Squamous Carcinoma Cells as 1, thereby doing binary classification and generating a 2×2 matrix for MobileNetV2 model. Fig. 15 shows the MobileNetV2 model confusion matrix.

The sensitivity of accuracy and the positivity of these two cells were 677 normal cells, 584 cancerous cells, 30 normal cells incorrectly classified as cancerous, and 74 cancerous cells misclassified as normal cells.

With low False Positives 30 and False Negatives 74, and high True Negatives 677 and True Positives 584, the model demonstrates its good performance and seldom cell misclassification.

MobileNet-V2 has shown better performance compared to the previous models, with lower misclassification rates. Unfreezing more layers and further fine-tuning the dataset could help the model to learn more data-specific features.

**Performance of Inception V3:**

**Fig. 16:**
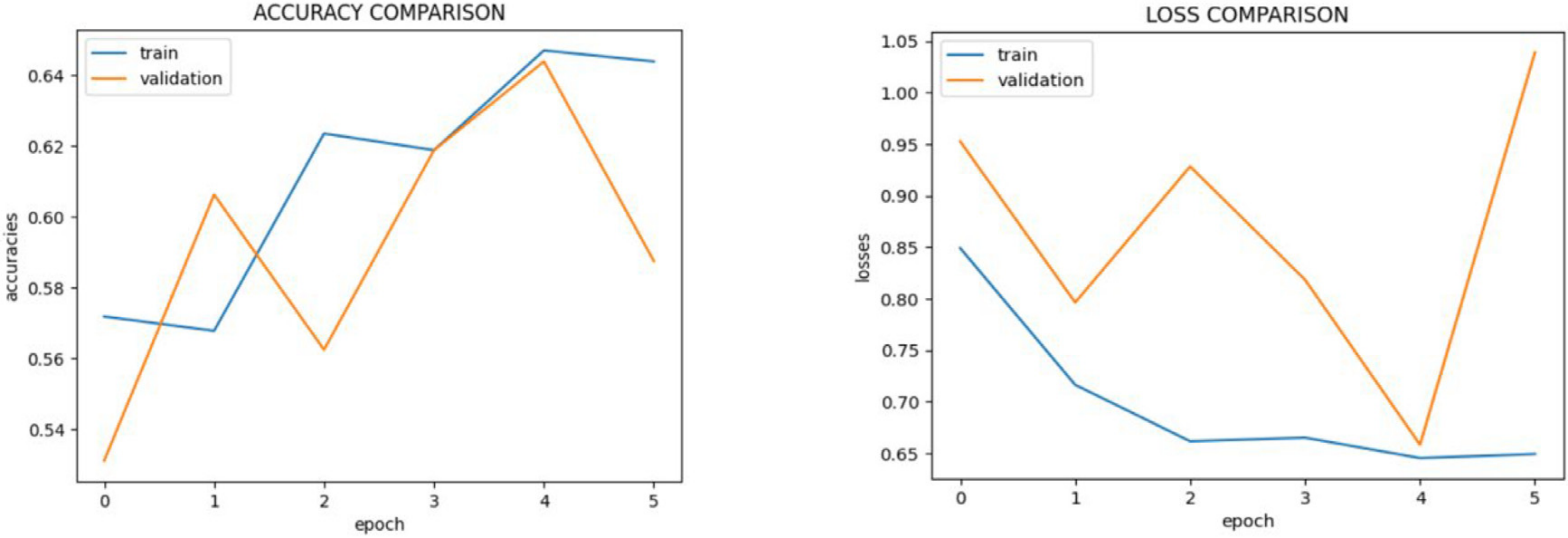
Inception V3 model performance metrics.

The Inception V3 model, which is well-known for its utilization of computational power and multi-scale feature extraction, successfully classified images into normal and malignant groups with an accuracy of 51.86 %. Fig. 16 shows the Inception V3 model performance metrics. This reflects the model's performance in handling diverse visual aspects with its intricate design.

The accuracy of Inception V3 model, when applied to the supplemented dataset, is found to be very less. The collection of photographs includes 100X and 400X magnification with normal tissue and oral squamous cell cancer. After supplementation, there were 1615 photos at 100X magnification for OSCC, 1815 normal photos at 400X magnification, and 1780 normal shots at 100X magnification. This augmentation might not have been enough to remove the bias in training because of the huge class imbalance between the normal and OSCC images in the original dataset. There could also have been anomalies or recurring patterns introduced by these augmentation methods that wouldn't generalize well for new data. The low performance could have been exacerbated by the Inception V3 model, too.

**Confusion Matrix:**

**Fig. 17:**
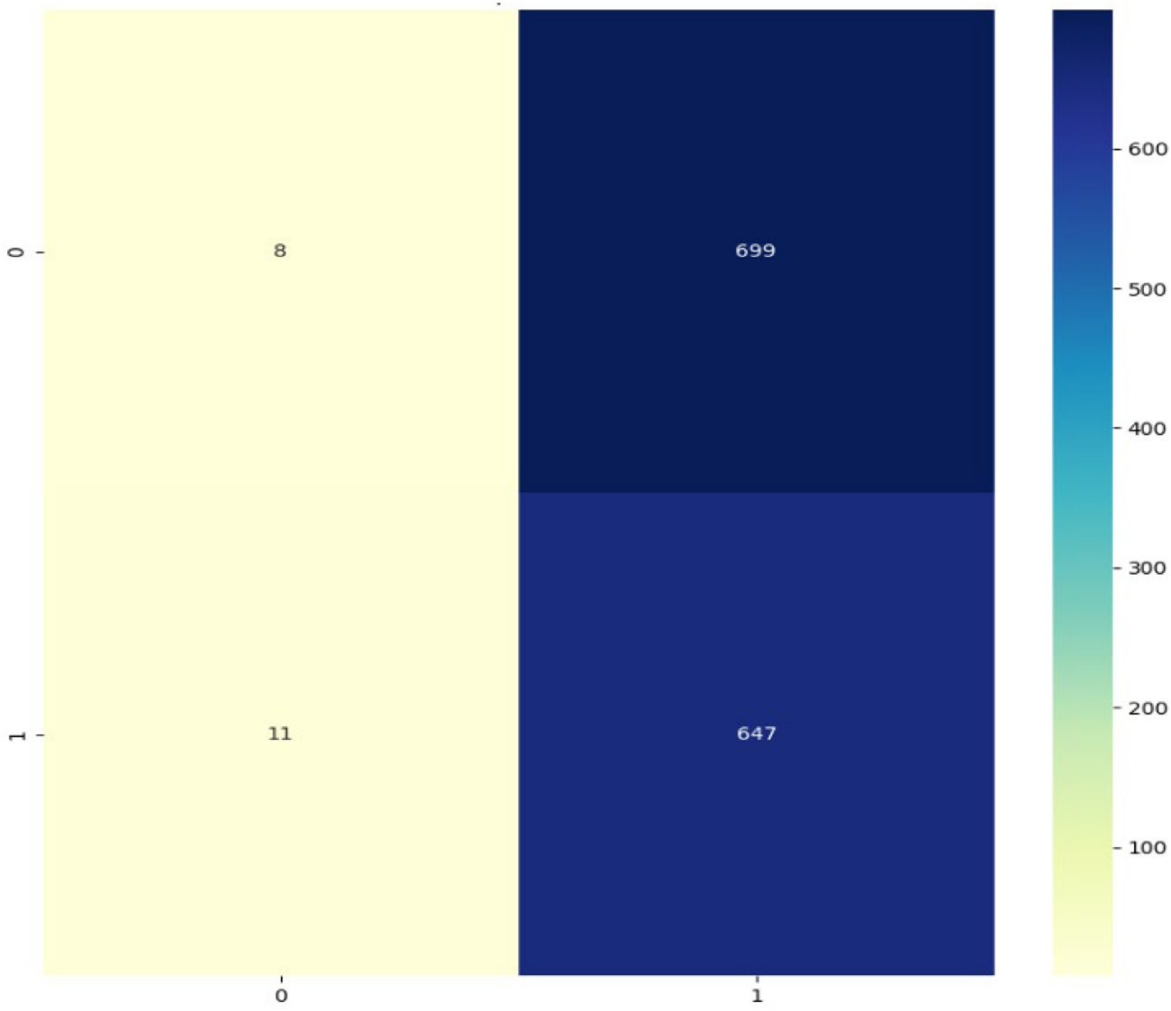
Inception V3 model confusion matrix.

The resulting confusion matrix performs a binary classification, producing a 2 × 2 matrix for the Inception V3 model, in which the normal cells are labeled as 0 and oral squamous carcinoma cells are labeled as 1. Fig. 17 shows the Inception V3 model confusion matrix.

Only 8 normal cells are accurately classified by the model, however it performs well when dealing with malignant cells 647. It wrongly labels a significant portion of normal cells 699 as malignant, whereas only a small percentage of malignant cells 11 are mistakenly classified as normal.

There is a high count which may have a great bias toward the cancer class. It may have outweighed the malignant characteristic when it decided and misclassified almost all of the normal cells as cancerous.

The model satisfactorily identifies most malignant cells, leaving room for increasing sensitivity towards wrongly classified samples. The InceptionV3 model for high bias to detect cancerous cells results in very high False Positives, which could be due to the fact that it requires an adjustment from class balance.

With an accuracy of 95.41 %, the MobileNetV2 model was found to be the most accurate of the models that were used. This model also surpassed all the implemented models in other evaluation metrics such as precision, recall, and F1-score. As a result, it is more appropriate for any clinical application where an accurate diagnosis made in a short interval of time is critical. The MobileNetV2 model is compared, using a variety of methods, to earlier studies carried out in similar fields in order to more clearly ascertain its efficiency. This study has consequently revealed the advantages of MobileNetV2 in detecting squamous cell carcinoma in the oral cavity, which can be exploited to improve patient outcomes. Table 2 shows the model comparison study using evaluation metrics, which highlights MobileNetV2’s superior performance.

**Table 2:** Model comparison study using evaluation metrics.

Measures	Vgg-16	ResNet50	LeNet-5	MobileNetV2	Inception V3
**Accuracy (%)**	76.54	71.00	76.56	95.41	51.86
**Precision (%)**	74.90	76.12	87.00	95.03	51.29
**Recall (%)**	78.99	60.43	77.00	89.06	99.21
**F1-Score (%)**	76.89	67.28	81.47	92.08	67.62

Each of the inverted residual blocks that make up the MobileNetV2 model has three layers. The model can effectively learn more complicated features owing to this inverted. As an outcome it can be said that MobileNetV2 set a new standard for accuracy when compared to previous research and demonstrated to be the most suitable model for histopathological oral cancer identification. Another element contributing to the performance was the augmentation strategy used to expand the dataset from 1224 photos to 6931 images, allowing the model to learn with greater precision than in previous studies. A large dataset and advanced deep learning techniques ensure that the results are accurate for enhanced diagnostic skills in any clinical setting, thereby enhancing patient care and treatment outcomes. Table 3 presents a comparative performance metrics analysis, further illustrating MobileNetV2’s performance advantages over other models.

**Table 3:** A comparative performance metrics.

Previous Studies	Model	Accuracy (%)	Precision (%)	Recall (%) Sensitivity	F1-Score (%)
[24]	DenseNet201	91.25	93.00.	93.00	93.00
[25]	Proposed Model	90.02	–	–	90.15
[26]	Decision Tree	70.59	–	41.98	–
[27]	MobileNet	71.5	0.47	–	–
**(Our Best Model)**	MobileNetV2	95.43	89.06	92.08	

While there are various approaches for identifying oral cancer, histopathological image analysis stands out because it can identify malignant cells even in the early stages. This is feasible considering these images examine cells at a microscopic level to detect abnormalities in their structure, such as uneven cell shape, accelerated cell division, and invasion of adjacent tissues. Deep learning algorithms can help doctors diagnose oral cancer more effectively. The manual diagnosis method largely depends on the competence and knowledge of the physicians because it takes time to identify every tissue in the biopsy that was taken from the patient. Despite this, problems with manual diagnosis persist among doctors regarding the diagnosis [7]. This issue is addressed by utilizing AI-powered approaches. Deep learning models are trained on a very large quantity of data, applying identical standards to every image, thereby lowering the chance of human error.

Additionally, these deep learning models can process images relatively quicker than pathologists, who may take longer. These models not only increment diagnostic accuracy and speed, but they also offer assistance the specialists by guiding the course of treatment, eliminating diagnostic biases, and helping healthcare systems in regions with limited resources. The comparative analysis of the models VGG-16, ResNet50, LeNet5, MobileNetV2, and InceptionV3 indicated that MobileNetV2 achieved the maximum accuracy of 95.41 %, leading to the conclusion that it is the most efficient model for detecting oral cancer from squamous cell carcinoma.

### Limitations

Considering the model complexities, this work had a few limitations with respect to its major dataset of 6391 images. Basically, increasing the volume of data enhances the model complexity, but the challenge is maintaining optimal performance. The more complicated these models get to be able to handle this enormous dataset, the risk of overfitting or inefficiency in computations impairs the overall accuracy and generalization capability of the algorithms.

Being that the normal class is the most underrepresented among the classes, class balancing techniques range from adjusting the weights of classes to using oversampling and under-sampling techniques in order to reduce imbalance. By adding more variety and volume to the training set for the normal class, data augmentation may enhance the model's recognition performance[28] Furthermore, regularization methods like dropout or L2 regularization, in conjunction with model tuning—which includes modifying parameters or investigating different architectures—could lessen overfitting and enhance validation performance. Optimizing the rate of learning could potentially enhance the convergence and overall performance of the model [29].

## Ethics statements

None

## CRediT authorship contribution statement

**Prerna Kulkarni:** Methodology, Software. **Nidhi Sarwe:** Conceptualization, Writing – original draft, Writing – review & editing. **Abhishek Pingale:** Data curation, Writing – review & editing. **Yash Sarolkar:** Project administration, Supervision. **Rutuja Rajendra Patil:** Conceptualization, Validation, Supervision. **Gitanjali Shinde:** Validation. **Gagandeep Kaur:** Validation.

## Declaration of competing interest

The authors declare that they have no known competing financial interests or personal relationships that could have appeared to influence the work reported in this paper.

## Data Availability

I have shared the link to my Dataset used and the code in Resource Availabililty Section in the Specification Table.
